# Differences in trunk accelerometry between frail and non-frail elderly persons in functional tasks

**DOI:** 10.1186/1756-0500-7-100

**Published:** 2014-02-21

**Authors:** Alejandro Galán-Mercant, Antonio I Cuesta-Vargas

**Affiliations:** 1 Physiotherapy Department, Faculty of Health Sciences, IBIMA, Universidad de Malaga, Av/Arquitecto Peñalosa s/n (Teatinos Campus Expansion), 29009 Málaga, Spain; 2 School of Clinical Sciences of the Faculty of Health at the Queensland University of Technology, Brisbane, Australia

**Keywords:** Frailty syndrome, Expanded timed-get-up-and-go test, iPhone, Inertial sensor

## Abstract

**Background:**

Physical conditions through gait and other functional task are parameters to consider for frailty detection. The aim of the present study is to measure and describe the variability of acceleration, angular velocity and trunk displacement in the ten meter Extended Timed Get-Up-and-Go test in two groups of frail and non-frail elderly people through instrumentation with the *iPhone4®* smartphone. Secondly, to analyze the differences and performance of the variance between the study groups (frail and non-frail).

This is a cross-sectional study of 30 subjects aged over 65 years, 14 frail subjects and 16 non-frail subjects.

**Results:**

The highest difference between groups in the Sit-to-Stand and Stand-to-Sit subphases was in the y axis (vertical vector). The minimum acceleration in the Stand-to-Sit phase was -2.69 (-4.17 / -0.96) m/s^2^ frail elderly versus -8.49 (-12.1 / -5.23) m/s^2^ non-frail elderly, p < 0.001. In the Gait Go and Gait Come subphases the biggest differences found between the groups were in the vertical axis: -2.45 (-2.77 /-1.89) m/s^2^ frail elderly versus -5.93 (-6.87 / -4.51) m/s^2^ non-frail elderly, p < 0.001. Finally, with regards to the turning subphase, the statistically significant differences found between the groups were greater in the data obtained from the gyroscope than from the accelerometer (the gyroscope data for the mean maximum peak value for Yaw movement angular velocity in the frail elderly was specifically 25.60°/s, compared to 112.8°/s for the non-frail elderly, p < 0.05).

**Conclusions:**

The inertial sensor fitted in the *iPhone4®* is capable of studying and analyzing the kinematics of the different subphases of the Extended Timed Up and Go test in frail and non-frail elderly people. For the Extended Timed Up and Go test, this device allows more sensitive differentiation between population groups than the traditionally used variable, namely time.

## Background

Clinical frailty syndrome is a common geriatric syndrome which is characterized by physiological reserve decreases and increased vulnerability and which may, in the event of unexpected intercurrent processes, result in falls, hospitalization, institutionalization or even death [1]. The changes associated to ageing and frailty bring changes in gait characteristics and the basic functional capacities of the individual [2]. This variability in different movement patterns has been interpreted as a more conservative gait pattern in order to increase stability and reduce the risk of falls [3]. The new, more conservative gait pattern has greater cognitive involvement and produces a result focused entirely on movement, whilst the perception of unexpected trigger factors may be overlooked [4]. Dual tasks have been shown to affect normal gait development even in non-frail persons [5].

The Timed Get-Up-and-Go (TGUG) test is a widely used tool to evaluate balance and some functional tasks through clinical evaluation of mobility and the risk of falls [2,6-8]. The clinical potential of the TGUG test comes from the sequencing of several basic functional abilities such as standing up and sitting down transitions, transitions which require balance, such as turning, and walking in a straight line [9]. These five sub-phases are common day-to-day activities and are often associated with falls [10]. The TGUG test, despite being widely used in clinical practice, has a series of limitations. The main limitations are: 1) It focuses only on the time variable and does not take into account other variables related to deficits in kinematics and kinetics which may affect balance or the risk of fall. 2) It measures the total time to perform the test, without taking into account partial times in the different functional tasks which make up the TGUG [11,12]. The TGUG test is currently carried out in an instrumented manner by attaching inertial sensors to the body [2,7,12-16].

The latest generation of smartphones often includes inertial sensors with subunits such as accelerometers and gyroscopes which can detect acceleration and inclination [17]. The numerous applications developed for these smartphones mean the data from the accelerometer and the gyroscope can be read, stored, transferred and displayed [18,19]. These applications evaluate and assess kinematic variables related to gait [20], measuring Cobb angles in x-rays, or provide an objective method to classify levels of physical activity and give an indication of the degree of functional capacity and quality of life [17,21].

The hypothesis of the study is that it is feasible to evaluate the differences between the frail and non-frail through a functional evaluation instrumented kinematically. The goals of the present study are as follows. Firstly, to measure and describe the magnitude of acceleration, angular velocity and trunk displacement in the ten meter Extended Timed Get-Up-and-Go (ETGUG) test in two groups of frail and non-frail elderly people through instrumentation with the *iPhone4®* smartphone. Secondly, to analyze the performances and differences between the study groups (frail and non-frail).

## Methods

### Design and participants

A cross-sectional study that involved 30 subjects aged over 65 years, 14 frail and 16 non-frail subjects. The participants were classified with frail syndrome by the Fried criteria (unintentional weight loss, self-reported exhaustion, weakness, slow walking speed, and low physical activity) [1]. The inclusion criterion was anyone aged over 65 years who does not present any of the exclusion criteria described in the study. Exclusion criteria were no history of pain in the last twenty-four hours, previous surgery, presence of a tumor or musculoskeletal disorders in the upper or lower extremity. Patients with impaired cognition, musculoskeletal back co-morbidities and problems associated to exercise intolerance were also excluded. All participants were clinically examined by a physiotherapist, and none of them were found to have any exclusion criteria. Table 1 shows the characteristics of the sample and stopwatch values in the ETGUG test.

**Table 1:** Characteristics of sample (N = 30)

	**Mean**		**SD**	
	**Frail (n = 14)**	**Non-frail (n = 16)**	**Frail (n = 14)**	**Non-frail (n = 16)**
Age (years)	83.71	70.25	6.37	3.32
Mass (kg)	56.21	71.03	9.64	13.11
Height (cm)	155.79	159.44	7.81	10.61
Body mass index (kg/m^2^)	23.36	27.87	3.48	3.79
Total Score ETGUG (s)	53.64	15.52	24.12	2.91

Kg, kilograms; cm, centimetres; m, metres; s, seconds.

Non-frail participants were recruited through advertisements at the Sport and Health Centre in Torremolinos, Spain. Frail participants were recruited through advertisements in Geriatrics Centers in Torremolinos and Benalmadena, Spain. Written informed consent was obtained from each individual. The study was approved by the ethics committee of the Faculty of Medicine at the University of Malaga, Spain.

### Expanded timed-get-up-and-go test

All subjects performed the ETGUG test three times and the best trial was selected based on the total score to complete the full test. Devices were not removed between trials. Subjects had five recess minutes between trials. All subjects used an armless chair and were instructed not to use their arms to stand up. Although an armchair is used in traditional ETGUG [22], an armless chair was used in our test. Previous studies have explored using armless chairs [23,24]. Using armless chairs could reduce variability between subjects by eliminating the choice to use or not use the armrests to arise [11]. ETGUG test used a 10 meter walkway to include more gait cycles during the test [24]. The beginning and the end of the walkway were marked with 2.5 cm green tape on the floor. The tape markings were shown to the subjects before the trials. Subjects were instructed to sit straight with their backs touching the back of the chair. After they were given the go signal by the tester, they arose from the chair, walked at their fastest walking speed but without running, turned right or left after passing the green tape at the end of the way, returned to the start chair, turned around and sat down. The tester timed their performance with a stopwatch.

### Phases of the expanded timed-get-up-and-go test

Offline data processing was used for identification of the different phases of the ETGUG test, divided into five phases: Sit-to-Stand (Si-St), Gait-Go (GG), Turning (T), Gait-Come (GC) and Turn-to-Stand-to-Sit (T-St-Si). Each phase of the ETGUG test was detected with acceleration data of the iPhone 4® accelerometer: Si-St and T-St-Si transitions were detected and analyzed using a method published elsewhere [25], and T transition was detected and analyzed using another method published elsewhere [7].

### Data collection and procedures

Linear acceleration was measured along three orthogonal axes using the iPhone 4® accelerometer snugly secured to the test subjects by a neoprene fixation belt over the sternum, with the smartphone screen facing forward. Previous studies show that the essential spatio-temporal characteristics of overground walking can be obtained by trunk accelerometry, individual step or stride cycles can be identified, and fair estimations of step length and walking speed can be obtained using a tri-axial accelerometer [26].

**Figure 1:**
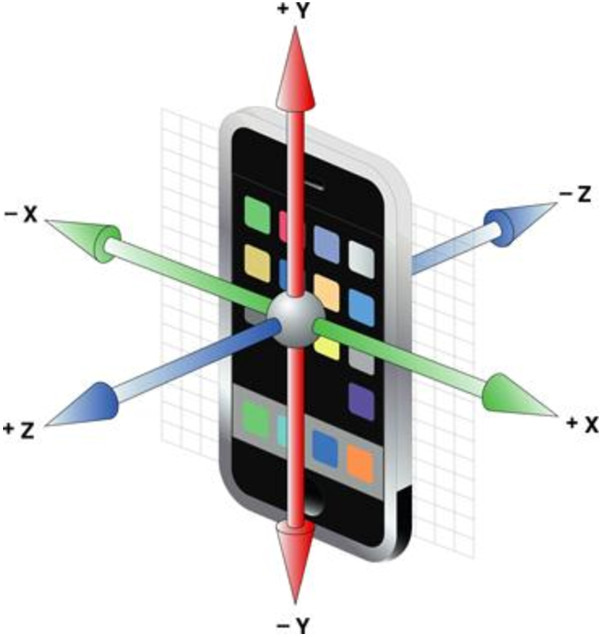
Orientation of the smartphone in the trunk.

The orientation and movement of the sensors are presented as RPY (roll, pitch, and yaw) Euler angles. If the sensor’s RPY axes are aligned with the anatomical axes of the trunk, the roll angle of a movement is around the anteroposterior (AP) axis, the pitch angle is around the left-right axis, and the yaw angle is around the vertical (V) axis (see Figure 1).

Apple® used a trialxial gyroscope, an accelerometer and a magnetometer in the iPhone 4® [27]. The application used to obtain kinematic data was *xSensor® Pro, Crossbow Technology, Inc.*, available in the *AppStore*, Apple®. The iPhone 4® has a storage capacity of 20 MB, and the data for each trial was transmitted as email for analysis and post-processing. The data sampling rate was set to 32 Hz. An iPhone 4® is required in order to obtain data jointly from the accelerometer, gyroscope and magnetometer, as earlier versions do not allow this possibility. A previous study showed an inter-observer error (standard deviation of the difference between measurements by two different observers) of 4.0° for the iPhone and 3.4° for the protractor [17].

### Data processing

Computerized automatic analysis was developed to filter the inertial sensor data and timing of sub-phases. This analysis was designed to systematically obtain kinematic data for further statistical analysis, and was performed using basic software package *R*®. Automatic analysis was guided in order to obtain independent kinematic information from the accelerometer and gyroscope, along with the timing for each subject in each of the five phases of the ETGUG test. The following was obtained from the accelerometer: maximum peak, minimum peak, mean and SDs of accelerations in the three movement axes (*x*, *y* and *z*). Furthermore, the maximum peak, minimum peak, mean and SDs of the resultant vector (RV) accelerations (RV = √ *x*2 + *y*2 + *z*2) were obtained. The following was obtained from the gyroscope: maximum peak, minimum peak, mean and SDs of rotation motions in the three movement axes (*x*, *y* and *z*). Finally*,* the following was obtained: maximum peak, minimum peak, mean and SDs of the angular velocity in the three movement axes (*x*, *y* and *z*).

### Statistical analysis

Analysis was performed with SPSS version 15 for Windows, while the data collection phase used inferential analysis between variables by type and normal. Non-parametric Mann–Whitney tests were used as determined by the normality of distribution variables. The statistical significance level was set at p < 0.05. Cohen’s *d* was used to estimate the magnitude of the relationship between differences. Cohen’s *d* is an effect size used to indicate the standardized difference between two means [28].

## Results

Tables 2, 3, 4 summarize the acceleration-based measures of the ETGUG test in the two groups. Stopwatch-based ETGUG duration showed higher duration for frail patients compared to the non-frail control group, as expected. This trend was significant in four phases (GG, T, GC, T-St-St). Other acceleration-based measures of amplitude are summarized in Tables 2, 3, 4.

**Table 2:** Acceleration-based values from the Si-St and T-St-Si phases (N = 30)

	**Frail (n = 14)**	**Non-frail (n = 16)**	**U**	**d**	**p-Value**
	**Median (IQR)**	**Median (IQR)**			
**Si-St**					
t.stopwatch (s)	7.03 (5.14 / 10.88)	2.23 (2.01 / 2.49)	13.00	1.255	<0.001
x.acc.max (m/s^2^)	2.11 (1.64 / 2.41)	3.43 (2.06 / 4.03)	49.50	-1.149	0.009
x.acc.min (m/s^2^)	-1.08 (-1.88 / -0.69)	-2.94 (-3.81 / -2.06)	30.50	-3.801	<0.001
y.acc.max (m/s^2^)	2.89 (1.94 / 3.87)	6.09 (4.87 / 7.04)	15.00	-1.972	<0.001
y.acc.min (m/s^2^)	-1.47 (-2.35 / -0.59)	-6.29 (-7.88 / -3.55)	0.000	2.622	<0.001
y.acc.mean (m/s^2^)	0.47 (0.31 / 0.94)	0.01 (-0.39 / 0.48)	45.00	1.069	0.005
rv.acc.max ( m/s^2^)	6.41 (5.43 / 8.31)	8.49 (7.56 / 10.78)	58.00	-0.803	0.025
rv.acc.mean ( m/s^2^)	2.71 (2.11 / 3.64)	4.12 (3.67 / 4.89)	44.00	-1.222	0.005
**T-St-Si**					
t.stopwatch (s)	10.42 (7.64 / 14.52)	3.71 (2.88 / 4.01)	2.00	1.966	<0.001
y.acc.max (m/s^2^)	3.13 (2.23 / 3.92)	6.03 (5.01 / 7.68)	26.50	-1.389	<0.001
y.acc.min (m/s^2^)	-2.69 (-4.17 / -0.96)	-8.49 (-12.1 / -5.23)	14.00	1.723	<0.001
z.acc.max (m/s^2^)	5.94 (4.32 / 7.31)	2.45 (1.96 / 5.89)	62.00	0.818	0.038
z.acc.min (m/s^2^)	-2.99 (-6.13 / -2.52)	-6.88 (-8.51 / -4.34)	35.00	1.329	<0.001
z.acc.mean (m/s^2^)	0.57 (-0.12 / 2.28)	-1.44 (-3.34 / -0.16)	36.00	1.473	0.002
rv.acc.max ( m/s^2^)	7.11 (5.22 / 7.72)	10.65 (7.91 / 12.38)	41.00	-1.120	0.003
rv.acc.min ( m/s^2^)	0.28 (0.19 / 0.47)	0.71 (0.49 / 1.01)	38.00	-1.157	0.002
rv.acc.mean ( m/s^2^)	3.03 (2.73 / 3.81)	4.39 (3.24 / 5.27)	45.00	-1.202	0.005

x, x axis; y, y axis; z, z axis; acc, acceleration; t, time; max, maximum; min, minimum; rv, resultant vector; U, U-Mann–Whitney; Si-St, Sit to Stand; T-St-Si, Turn Stand to Sit; d, Cohen’s d; IQR, interquartil range (percentil 25% / percentil 75%).

**Table 3:** Acceleration-based values from the GG and GC phases (N = 30)

	**Frail (n = 14)**	**Non-frail (n = 16)**	**U**	**d**	**p-Value**
	**Median (IQR)**	**Median (IQR)**			
**GG**					
t.stopwatch (s)	10.23 (7.82 / 15.04)	3.71 (2.45 / 3.98)	0.000	2.119	<0.001
x.acc.max (m/s^2^)	2.06 (1.64 / 2.45)	5.84 (3.55 / 8.78)	14.00	-1.479	<0.001
x.acc.min (m/s^2^)	-2.45 (-2.77 /-1.89)	-5.93 (-6.87 / -4.51)	9.00	1.910	<0.001
y.acc.max (m/s^2^)	2.50 (2.25 / 3.04)	9.59 (7.81 / 10.89)	0.000	-5.212	<0.001
y.acc.min (m/s^2^)	-2.50 (-3.35 / -2.04)	-9.07 (-12.71 / -5.63)	13.00	1.578	<0.001
y.acc.mean (m/s^2^)	0.15 ( 0.09 / 0.22)	0.46 (0.29 / 1.13)	29.00	-1.234	0.002
z.acc.min (m/s^2^)	-1.96 (-3.95 / -1.08)	-7.64 (-0.48 / -6.52)	0.000	3.303	<0.001
z.acc.mean (m/s^2^)	-0.57 (-1.62 / 0.58)	-2.72 (-9.99 / -2.39)	18.00	1.936	<0.001
rv.acc.max ( m/s^2^)	4.03 (3.22 / 4.99)	11.71 (10.33 / 13.87)	0.000	-1.957	<0.001
rv.acc.min ( m/s^2^)	0.42 (0.14 / 0.85)	1.54 (0.97 / 2.47)	29.00	-1.258	0.002
rv.acc.mean ( m/s^2^)	1.91 (1.51 / 2.33)	6.03 (5.27 / 6.75)	0.000	-4.776	<0.001
**GC**					
t.stopwatch (s)	5.91 (4.54 / 10.45)	1.85 (1.55 / 2.21)	8.00	1.923	<0.001
x.acc.max (m/s^2^)	2.35 (1.83 / 2.62)	5.03 (3.54 / 7.35)	19.00	-1.587	<0.001
x.acc.min (m/s^2^)	-2.40 (-3.26 / -1.81)	-5.56 (-7.95 / -3.95)	13.00	1.859	<0.001
y.acc.max (m/s^2^)	2.35 (1.94 / 2.97)	9.26 (8.50 / 11.07)	0.000	-5.436	<0.001
y.acc.min (m/s^2^)	-2.59 (-3.38 / -2.06)	-8.25 (-15.62 / -5.43)	13.00	1.389	<0.001
y.acc.mean (m/s^2^)	0.18 (0.11 / 0.26)	0.38 (0.31 / 1.12)	24.00	-1.199	<0.001
z.acc.mean (m/s^2^)	-0.45 (-1.81 / 0.61)	-2.98 (-4.62 / -2.35)	17.00	2.024	<0.001
rv.acc.max ( m/s^2^)	4.31 (3.39 / 5.03)	11.85 (9.18 / 16.26)	0.000	-1.802	<0.001
rv.acc.min ( m/s^2^)	0.33 (0.21 /0.65)	1.53 (0.64 / 3.13)	30.00	-1.292	0.002
rv.acc.mean ( m/s^2^)	1.96 (1.49 / 2.64)	5.79 ( 5.31 / 6.97)	0.000	-4.859	<0.001

x, x axis; y, y axis; z, z axis; acc, acceleration; t, time; max, maximum; min, minimum; rv, resultant vector; U, U-Mann–Whitney; GG, Gait Go; GC, Gait Come; d, Cohen’s d; IQR, interquartil range (percentil 25% / percentil 75%).

**Table 4:** **Acceleration-based values from the T phase (*N*** **= 30)**

	**Frail (n = 14)**	**Non-frail (n = 16)**	**U**	**d**	**p-Value**
	**Median (IQR)**	**Median (IQR)**			
t.stopwatch (s)	10.42 (7.64 / 14.53)	1.83 (1.46 / 2.12)	2.000	1.441	<0.001
x.acc.min (m/s^2^)	-1.86 (-2.89 / -1.25)	-5.62 (-7.85 / -4.23)	41.00	2.014	0.003
y.acc.max (m/s^2^)	2.01 (1.47 / 2.87)	7.72 (4.64 / 9.74)	26.50	-2.629	<0.001
y.acc.min (m/s^2^)	-1.62 (-3.14 / -1.18)	-6.01 (-11.77 / -4.92)	14.00	1.503	<0.001
z.acc.min (m/s^2^)	-1.52 (-2.89 / -0.44)	-6.80 (-9.65 / -4.97)	35.00	2.604	<0.001
z.acc.mean (m/s^2^)	-0.08 (-1.29 / 0.93)	-2.57 (-3.84 / -1.89)	36.00	1.830	0.002
rv.acc.max ( m/s^2^)	3.48 (2.65 / 4.82)	10.34 (8.30 / 13.08)	41.00	-1.782	0.003
rv.acc.min ( m/s^2^)	0.39 (0.20 / 0.67)	1.23 (0.69 / 2.05)	38.00	-0.930	0.002

x, x axis; y, y axis; z, z axis; acc, acceleration; t, time; max, maximum; min, minimum; rv, resultant vector; U, U-Mann–Whitney; T, Turn; d, Cohen’s d; IQR, interquartil range (percentil 25% / percentil 75%).

Tables 5, 6, 7 summarize the gyroscope-based measures of the ETGUG test in the two groups.

**Table 5:** **Gyroscope-based values from the Si-St and T-St-Si phases (*N*** **= 30)**

	**Frail (n = 14)**	**Non-frail (n = 16)**	**U**	**d**	**p-Value**
	**Median (IQR)**	**Median (IQR)**			
**Si-St**					
t.stopwatch	7.03 (5.14 / 10.88)	2.23 (2.01 / 2.49)	13.00	1.255	<0.001
roll.rotation.max (deg)	162.4 (20.92 / 179.3)	196.54 (179.4 / 287.4)	35.00	-0.897	0.001
roll.rotation.mean (deg)	-38.62 (-62.62 / 46.11)	83.83 (-48.28 / 220.73)	61.00	-0.951	0.034
rate.yaw.min (deg/s)	-44.39 (-59.08 / -31.93)	-26.13 (-33.96 / -2.36)	42.00	-0.978	0.004
rate.pitch.max (deg/s)	22.56 (16.63 / 38.59)	123.40 (36.47 / 287.21)	28.00	-0.954	<0.001
rate.roll.max (deg/s)	17.68 (12.56 / 24.93)	165.43 (79.95 / 319.6)	0.000	-1.708	<0.001
rate.roll.min (deg/s)	-17.11 (-29.47 / -10.36)	-62.59 (-89.82 / -15.96)	56.00	1.482	0.020
rate.roll.mean (deg/s)	0.58 (-0.76 / 1.27)	49.99 (-0.43 / 134.31)	59,00	-0.852	0.028
**T-St-Si**					
t.stopwatch (s)	10.42 (7.64 / 14.52)	3.71 (2.88 / 4.01)	2.00	1.966	<0.001
roll.rotation.min (deg)	-176.1 (-179.27 / -166.93)	-49.25 (-178.4 / 0.31)	60.00	-1.254	0.031
roll.rotation.mean (deg)	-19.03 (-44.13 / 11.71)	30.81 (-28.32 / 247.87)	62.00	-0.942	0.038
rate.yaw.max (deg/s)	40.31 (34.54 / 51.77)	65.53 (36.13 / 292.39)	57.00	-0.908	0.022
rate.yaw.min (deg/s)	-67.38 (-87.12 / -46.83)	-33.68 (-63.23 / -4.24)	49.00	-1.151	0.009
rate.roll.max (deg/s)	32.95 (26.11 / 44.67)	80.42 (62.84 / 295.52)	13.00	-1.159	<0.001
rate.roll.min (deg/s)	-20.51 (-32.63 / -14.27)	-74.50 (-102.45 / -13.4)	58.00	1.185	0.025

max, maximum; min, minimum; t, time; s, second; deg, degrees; rate, angular velocity; U, U-Mann–Whitney; Si-St, Sit to Stand; T-St-Si, Turn Stand to Sit; d, Cohen’s d; IQR, interquartil range (percentil 25% / percentil 75%).

**Table 6:** Gyroscope-based values from the GG and GC phases (N = 30)

	**Frail (n = 14)**	**Non-frail (n = 16)**	**U**	**d**	**p-Value**
	**Median (IQR)**	**Median (IQR)**			
**GG**					
t.stopwatch (s)	10.23 (7.82 / 15.04)	3.71 (2.45 / 3.98)	0.000	2.119	<0.001
pitch.rotation.min (deg)	77.20 (74.98 / 80.62)	62.81 (56.76 / 67.66)	10.00	2.288	<0.001
pitch.rotation.mean (deg)	81.38 (80.10 / 85.91)	70.88 (61.50 / 75.95)	15.00	1.834	<0.001
rate.yaw.max (deg/s)	24.83 ( 21.53 / 30.63)	47.92 (39.05 / 291.91)	15.00	-0.984	<0.001
rate.pitch.max (deg/s)	37.41 (33.91 / 47.66)	84.10 (69.34 / 295.27)	11.00	-1.150	<0.001
rate.roll.max (deg/s)	24.98 (19.42 / 34.56)	89.24 (53.61 / 305.78)	0.000	-1.349	<0.001
rate.roll.min (deg/s)	-21.15 ( -29.98 / -16.27)	-59.82 (-87.25 / -10.7)	56.00	1.153	0.020
**GC**					
t.stopwatch (s)	5.91 (4.54 / 10.45)	1.85 (1.55 / 2.21)	8.00	1.923	<0.001
pitch.rotation.max (deg)	85.88 (82.42 / 89.55)	80.95 (68.87 / 86.01)	51.00	1.056	<0.001
pitch.rotation.min (deg)	76.40 (72.73 / 78.39)	66.46 (59.57 / 72.02)	28.00	1.544	<0.001
pitch.rotation.mean (deg)	81.47 (78.51 / 85.79)	73.05 (63.02 / 78.14)	30.00	1.469	<0.001
roll.rotation.min (deg)	-164.7 (-178.8 / -67.73)	-22.84 (-40.95 / -3.04)	39.00	-1.160	0.002
rate.yaw.max (deg/s)	25.11 (22.44 / 34.93)	45.37 (35.42 / 286.57)	21.00	-0.936	<0.001
rate.pitch.max (deg/s)	37.75 (30.13 / 46.51)	77.28 (70.53 / 298.20)	13.00	-0.903	<0.001
rate.roll.max (deg/s)	26.41 (21.11 / 36.16)	81.58 (52.24 / 293.82)	3.00	-0.715	<0.001
rate.roll.min (deg/s)	-23.70 (-36.51 / -16.71)	-50.66 (-66.75 / -10.6)	58.00	-1.303	0.025

max, maximum; min, minimum; t, time; s, second; deg, degrees; rate, angular velocity; U, U-Mann–Whitney; GG, Gait Go; GC, Gait Come; d, Cohen’s d; IQR, interquartil range (percentil 25% / percentil 75%).

**Table 7:** Gyroscope-based values from the T phase (N = 30)

	**Frail (n = 14)**	**Non-frail (n = 16)**	**U**	**d**	**p-Value**
	**Median (IQR)**	**Median (IQR)**			
t.stopwatch (s)	10.42 (7.64 / 14.53)	1.83 (1.46 / 2.12)	2.000	1.441	<0.001
roll.rotation.min (deg)	-177.55 (-179.25 / -174.8)	-53.57 (-83.94 / -0.41)	60.00	-2.57	0.031
roll.rotation.mean (deg)	-11.85 (-23.22 / 32.49)	63.35 (-11.76 / 152.64)	62.00	-0.939	0.038
rate.yaw.max (deg/s)	25.60 (19.70 / 30.83)	112.81 (23.06 / 285.92)	57.00	-0.825	0.022
rate.yaw.min (deg/s)	-22.79 (-32.53 / -19.05)	-52.89 (-78.17 / -11.56)	49.00	1.117	0.009
rate.roll.max (deg/s)	27.09 (13.78 / 31.72)	134.55 (49.80 / 295.59)	13.00	-1.131	<0.001
rate.roll.min (deg/s)	-18.97 (-26.13 / 12.74)	-39.88 (-59.05 / -5.59)	58.00	0.959	0.025

max, maximum; min, minimum; t, time; s, second; deg, degrees; rate, angular velocity; U, U-Mann–Whitney; T, Turn; d, Cohen’s d; IQR, interquartil range (percentil 25% / percentil 75%).

The highest difference between groups in the Sit-to-Stand and Stand-to-Sit subphases was in the y axis (vertical vector). The minimum acceleration in the Stand-to-Sit phase was -2.69 (-4.17 / -0.96) m/s^2^ frail elderly versus -8.49 (-12.1 / -5.23) m/s^2^ non-frail elderly, p < 0.001. In the Gait Go and Gait Come subphases the biggest differences found between the groups were in the vertical axis: -2.45 (-2.77 /-1.89) m/s^2^ frail elderly versus -5.93 (-6.87 / -4.51) m/s^2^ non-frail elderly, p < 0.001. Finally, with regards to the turning subphase, the statistically significant differences found between the groups were greater in the data obtained from the gyroscope than from the accelerometer (the gyroscope data for the mean maximum peak value for Yaw movement angular velocity in the frail elderly was specifically 25.60°/s, compared to 112.8°/s for the non-frail elderly, p < 0.05).

## Discussion

The present study has described and examined the identification, analysis and differentiation in the performance of kinematic variables using the inertial sensor fitted in the *iPhone4®* during the subphases of the ETGUG test in non-frail and frail elderly persons. Significant differences were found between the groups of elderly persons in the accelerometry and angular displacement variables obtained in the kinematic readings of the trunk during the subphases of the ETGUG test.

The results obtained in this study show lower values in the frail elderly population group. The most significant differences found in the Si-St subphase corresponded to accelerometry, with the frail elderly persons obtaining lower minimum accelerations than the non-frail elderly people in the y axis. The most significant differences found in the T-St-Si subphase corresponded to accelerometry, with the frail elderly persons obtaining lower minimum accelerations than the non-frail elderly people in the y axis during these phases. In the GG and GC subphases the greatest differences found between the groups were in the y axis (maximum accelerations). Finally, with regards to accelerometry in the turning subphase, the greatest differences found between the groups were in the y axis (maximum accelerations).

As far as we are aware, this is the first study which has used *iPhone4®* technology to analyze and study the kinematics of non-frail and frail persons aged over 65 years during the ETGUG test. Moreover, it is the first study which has shown the possibility of differentiating kinematic patterns in the subphases of the ETGUG. The instrumented kinematic analysis of the Timed Get-Up-and-Go test was analyzed previously [23]. However, unlike the present study, other tests did not use the extended Timed Get-Up-and-Go test, which presents more analyzable gait cycles during the GG and GC phases [24]. Moreover, no data were provided regarding magnitudes of acceleration and angular velocity or their duration, nor were there any results regarding the subphases of the traditional Timed Get-Up-and-Go. The main advantage of the instrumentation of the ETGUG test is that it allows detailed readings of multiple variables in each of the subphases of the test. By way of example, the results of the present study obtained in Table 2 show kinematic data which inform us that in the Si-St phase the linear acceleration of the trunk on the y axis showed significant differences between non-frail and frail elderly persons, whilst linear acceleration in the z axis did not show any statistically significant differences.

It should be noted that frailty is defined as a clinical syndrome in which three or more of the following criteria should be present: unintentional weight loss, self-referred exhaustion, muscular weakness, low walking speed and low physical activity levels [1]. Generically, the gyroscope and accelerometry data obtained for the Si-St and T-St-Si transitions were similar to other studies with other types of study group. In the present study, the frail elderly showed low values in the kinematic variables compared to the controls, the same as the subjects affected by Parkinson’s disease [11,16,29], the elderly with a high risk of falls [2] and the frail elderly in a previous study [14].

Three recent studies [11,12,29] have instrumented the Timed Get-Up-and-Go test, differentiating and analyzing the kinematic data in each of the five subphases of the test (Si-St, GG, T, GC, T-St-Si) between two groups of elderly persons. However, unlike the present study, they did not use *iPhone4®* technology to collect kinematic variables. Their goal was to differentiate movement patterns for elderly persons with Parkinson’s disease, carrying out the tests over a distance of seven meters.

Another recent study which has worked on the instrumentalization of the Timed Get-Up-and-Go [2] test systematically evaluated the accelerometry values in elderly persons with a high risk of falls during the traditional three meter test, focusing solely on transitions in Si-St and T-St-Si. Like the present study, this study found numerous variables deriving from acceleration which showed differences between groups. However, the variables in this study were different, as was the methodology, etc. Moreover, the measurement units were not the same, and this study was based on acceleration increase amplitude and acceleration slope [2]. From a clinical perspective, the present study demonstrates that these new accelerometry parameters play an important role in differentiating between subjects with different functional states. These results provide new knowledge, extending existing knowledge on the isolated study of Si-St and T-St-Si transitions in frail and non-frail elderly people [13,14].

With regards to analysis of the data obtained in the present study, the differences between the frail and the physically active elderly show a series of lower values in the group of frail persons in each of the five subphases which make up the ETGUG test. It is notable that the lower value for the frail elderly in the Si-St and t-St-Si subphase corresponded to accelerometry, with the frail elderly obtaining much lower minimum and maximum accelerations than the physically active elderly in the y axis (see Table 2) during these phases. In kinematic terms, this axis corresponds to accelerations in the VT axis, leading us to believe that the frail elderly have less strength to carry out the impulse in concentric contraction of the quadriceps femoris muscle and the decrease in eccentric contraction of the same muscle on the VT axis, as required for the transition from sitting to standing and vice versa. Increased leg strength, leg power and overall balance can improve mobility and reduce the risk of fall. Sensor-based assessment of peak power and acceleration during the sit-to-stand transfer may be useful for detecting changes in mobility and fall risk. Standard clinical tests as well as sensor-based measures of peak power and acceleration, maximal velocity and duration of normal and fast sit-to-stand showed significant improvements after a leg strengthening program. There is a significant direct relationship between leg strength, kinematic acceleration variables, kinematic velocity variables and stand-to-sit transfer performance [30]. A study of the factors which influence this transition in 669 institutionalized elderly people showed that quadriceps strength is the most important determinant factor for this transition, although there are other factors such as proprioception, movement execution speed and psychological aspects which also influence ability to successfully carry out this functional test. Ability to carry out this transition is probably also influenced by other motor skills of the individual, such as intramuscular and intermuscular coordination, space-time coordination, etc. [31]. Other factors which may influence this transition are foot position, anthropometry of the individual and chair height [32].

The largest differences found between the groups in the GG and GC subphases were in the three axes (see Table 3 and Table 6). As with other authors [14], the results of the present study indicate that the differences obtained in the Medio Lateral (ML), Vertical (VT) and Antero Posterior (AP) axes showed significant differences between the group of frail elderly and the controls. This could be due to variability of trunk movements during gait playing an active role in dynamic balance whilst walking. Future studies would be required in order to research sensitivity to detect differences between groups of acceleration during gait in all three motion axes.

With regards to the turning subphase, a previous study which analyzed the behavior of kinematic variables during turning in persons suffering from Parkinson’s disease [7] did not find statistically significant differences between the groups, save for the duration of the transition. However, the present study found statistically significant differences between groups in the aforementioned variables.

The results obtained open up the way for further research in the future, although this study presents a series of limitations: men and women have different characteristics, and it would be interesting to analyze differences in the kinematic data by gender in the ETGUG test. It would be interesting in futures studies to analyze the predictive capability of the kinematic variables which showed statistically significant differences in the different subphases of the ETGUG test between non-frail and frail elderly persons. This will help not only to understand which variables are of interest and are associated to identifying the frail elderly, but will also allow early differentiation of possible frail elderly, which may be of use in prevention in clinical practice. Additional work is also needed to explore other accelerometer and gyroscope-derived properties of the ETGUG test, including comparison with a gold standard.

## Conclusions

The inertial sensor fitted in the *iPhone4®* is able to study and analyze the kinematics of the different subphases of the ETGUG test in frail and non-frail elderly people. The accelerometry values for the frail elderly are lower than for the non-frail elderly. This suggests that the frail elderly carry out the test in a more careful, restricted way during the functional tasks which make up the ETGUG test, possibly showing their reduced ability to regulate movement when performing these tasks and transitions. These results indicate that the additional, relevant information for future discriminant analysis comes mainly from the acceleration signal during the ETGUG test. From a clinical perspective, the present study demonstrates that these new accelerometry parameters play an important role in differentiating between subjects with different functional states. These results provide new knowledge, extending existing knowledge of the isolated study of functional task in frail and non-frail elderly people [13,14,33,34].

## Competing interests

The authors declare that they have no competing interests.

## Authors’ contributions

AICV conceived of the study, participated in its design and coordination, and drafted the manuscript. AICV and AGM performed the statistical analysis and provided critical content revision of the manuscript. Both author’s read and approved the final manuscript. AICV had full access to all of the data in the study and takes responsibility for the integrity of the data and the accuracy of the data analysis.

## References

[B1] Fried LP Tangen CM Walston J Newman AB Hirsch C Gottdiener J Seeman T Tracy R Kop WJ Burke G McBurnie MA Frailty in older adults J Gerontol A Biol Sci Med Sci 2001 56 M146 M157 10.1093/gerona/56.3.M146 11253156

[B2] Weiss A Herman T Plotnik M Brozgol M Giladi N Hausdorff JM An instrumented timed up and go: the added value of an accelerometer for identifying fall risk in idiopathic fallers Physiol Meas 2011 32 2003 2018 10.1088/0967-3334/32/12/009 22094550

[B3] Menz HB Lord SR Fitzpatrick RC Age-related differences in walking stability Age Ageing 2003 32 137 142 10.1093/ageing/32.2.137 12615555

[B4] Lamoth CJ Van Deudekom FJ Van Campen JP Appels BA De Vries OJ Pijnappels M Gait stability and variability measures show effects of impaired cognition and dual tasking in frail people J Neuroeng Rehabil 2011 8 2 10.1186/1743-0003-8-2 21241487 PMC3034676

[B5] Beauchet O Dubost V Herrmann FR Kressig RW Stride-to-stride variability while backward counting among healthy young adults J Neuroeng Rehabil 2005 2 26 10.1186/1743-0003-2-26 16095533 PMC1190208

[B6] Berg KO Maki BE Williams JI Holliday PJ Wood-Dauphinee SL Clinical and laboratory measures of postural balance in an elderly population Arch Phys Med Rehabil 1992 73 1073 1080 1444775

[B7] Salarian A Zampieri C Horak FB Carlson-Kuhta P Nutt JG Aminian K Analyzing 180° turns using an inertial system reveals early signs of progress in Parkinson’s Disease Conf Proc IEEE Eng Med Biol Soc 2009 2009 224 227 19964471 10.1109/IEMBS.2009.5333970 PMC2954632

[B8] Whitney SL Marchetti GF Schade A Wrisley DM The sensitivity and specificity of the Timed “Up & Go” and the Dynamic Gait Index for self-reported falls in persons with vestibular disorders J Vestib Res 2004 14 397 409 15598995

[B9] Rogers MA Phillips JG Bradshaw JL Iansek R Jones D Provision of external cues and movement sequencing in Parkinson’s disease Mot Control 1998 2 125 132 10.1123/mcj.2.2.125 9644283

[B10] Tinetti ME Speechley M Ginter SF Risk factors for falls among elderly persons living in the community N Engl J Med 1988 319 1701 1707 10.1056/NEJM198812293192604 3205267

[B11] Salarian A Horak FB Zampieri C Carlson-Kuhta P Nutt JG Aminian K iTUG, a sensitive and reliable measure of mobility IEEE Trans Neural Syst Rehabil Eng 2010 18 303 310 20388604 10.1109/TNSRE.2010.2047606 PMC2922011

[B12] Zampieri C Salarian A Carlson-Kuhta P Nutt JG Horak FB Assessing mobility at home in people with early Parkinson’s disease using an instrumented Timed Up and Go test Parkinsonism Relat Disord 2011 17 277 280 10.1016/j.parkreldis.2010.08.001 20801706 PMC2995832

[B13] Ganea R Paraschiv-Ionescu A Büla C Rochat S Aminian K Multi-parametric evaluation of sit-to-stand and stand-to-sit transitions in elderly people Med Eng Phys 2011 32 1086 1093 21601505 10.1016/j.medengphy.2011.04.015

[B14] Moe-Nilssen R Helbostad JL Interstride trunk acceleration variability but not step width variability can differentiate between fit and frail older adults Gait Posture 2005 21 164 170 10.1016/j.gaitpost.2004.01.013 15639395

[B15] Mellone S Tacconi C Chiari L Validity of a Smartphone-based instrumented Timed Up and Go Gait Posture 2012 36 163 165 10.1016/j.gaitpost.2012.02.006 22421189

[B16] Weiss A Herman T Plotnik M Brozgol M Maidan I Giladi N Gurevich T Hausdorff JM Can an accelerometer enhance the utility of the Timed Up & Go Test when evaluating patients with Parkinson’s disease? Med Eng Phys 2010 32 119 125 10.1016/j.medengphy.2009.10.015 19942472

[B17] Shaw M Adam CJ Izatt MT Licina P Askin GN Use of the iPhone for Cobb angle measurement in scoliosis Eur Spine J 2011 21 6 1062 1068 doi: 10.1007/s00586-011-2059-0. Epub 2011 Nov 9 22065167 10.1007/s00586-011-2059-0 PMC3366139

[B18] Mellone S Tacconi C Schwickert L Klenk J Becker C Chiari L Smartphone-based solutions for fall detection and prevention: the FARSEEING approach Z Gerontol Geriatr 2012 45 722 727 10.1007/s00391-012-0404-5 23184298

[B19] Tacconi C Mellone S Chiari L Smartphone-based applications for investigating falls and mobility Pervasive Computing Technologies for Healthcare (PervasiveHealth), 2011 5th International Conference on 2011 258 261

[B20] Lemoyne R Mastroianni T Cozza M Coroian C Grundfest W Implementation of an iPhone as a wireless accelerometer for quantifying gait characteristics Conf Proc IEEE Eng Med Biol Soc 2010 2010 3847 3851 21097067 10.1109/IEMBS.2010.5627699

[B21] Xia Y Cheung V Garcia E Ding H Karunaithi M Development of an automated physical activity classification application for mobile phones Stud Health Technol Inform 2011 168 188 194 21893928

[B22] Podsiadlo D Richardson S The timed “Up & Go”: a test of basic functional mobility for frail elderly persons J Am Geriatr Soc 1991 39 142 148 1991946 10.1111/j.1532-5415.1991.tb01616.x

[B23] Higashi Y Yamakoshi K Fujimoto T Sekine M Tamura T Quantitative evaluation of movement using the timed up-and-go test IEEE Eng Med Biol Mag 2008 27 38 4618270049

[B24] Wall JC Bell C Campbell S Davis J The Timed Get-up-and-Go test revisited: measurement of the component tasks J Rehabil Res Dev 2000 37 109 113 10847578

[B25] Najafi B Aminian K Loew F Blanc Y Robert PA Measurement of stand-sit and sit-stand transitions using a miniature gyroscope and its application in fall risk evaluation in the elderly IEEE Trans Biomed Eng 2002 49 843 851 10.1109/TBME.2002.800763 12148823

[B26] Dijkstra B Kamsma Y Zijlstra W Detection of gait and postures using a miniaturised triaxial accelerometer-based system: accuracy in community-dwelling older adults Age Ageing 2010 39 259 262 20083616 10.1093/ageing/afp249

[B27] LIS302DL accelerometer specs http://www.st.com/internet/analog/product/152913.jsp

[B28] Cohen J Statistical power analysis for the behavioral sciences 1987 Hillsdale, New Jersey: L. Erlbaum Associates

[B29] Zampieri C Salarian A Carlson-Kuhta P Aminian K Nutt JG Horak FB The instrumented timed up and go test: potential outcome measure for disease modifying therapies in Parkinson’s disease J Neurol Neurosurg Psychiatr 2010 81 171 176 10.1136/jnnp.2009.173740 19726406 PMC3065923

[B30] Regterschot GR Folkersma M Zhang W Baldus H Stevens M Zijlstra W Sensitivity of sensor-based sit-to-stand peak power to the effects of training leg strength, leg power and balance in older adults Gait Posture 2014 39 1 303 7 10.1016/j.gaitpost.2013.07.122 23973356

[B31] Lord SR Murray SM Chapman K Munro B Tiedemann A Sit-to-stand performance depends on sensation, speed, balance, and psychological status in addition to strength in older people J Gerontol A Biol Sci Med Sci 2002 57 M539 543 10.1093/gerona/57.8.M539 12145369

[B32] Janssen WGM Külcü DG Horemans HLD Stam HJ Bussmann JBJ Sensitivity of accelerometry to assess balance control during sit-to-stand movement IEEE Trans Neural Syst Rehabil Eng 2008 16 479 484 18990651 10.1109/TNSRE.2008.2003386

[B33] Zijlstra W Assessment of spatio-temporal parameters during unconstrained walking Eur J Appl Physiol 2004 92 39 44 10.1007/s00421-004-1041-5 14985994

[B34] Ganea R Paraschiv-Ionescu A Salarian A Büla C Martin E Rochat S Hoskovec C Piot-Ziegler C Aminian K Kinematics and dynamic complexity of postural transitions in frail elderly subjects Conf Proc IEEE Eng Med Biol Soc 2007 2007 6118 6121 18003411 10.1109/IEMBS.2007.4353745

